# Effectiveness and Safety of Zercepac and Reference Trastuzumab in the Neoadjuvant Setting for Early-Stage Breast Cancer: A Retrospective Cohort Study

**DOI:** 10.1155/2022/9998114

**Published:** 2022-11-03

**Authors:** Zheying Liu, Yinan Guan, Yongzhong Yao, Weijie Zhang, Xiaoming Zhuang, Yin Zhang, Tingting Zhu

**Affiliations:** ^1^Department of Breast Surgery, Southeast University School of Medicine, Nanjing 210009, China; ^2^Department of Breast Surgery, Jiangbei International Hospital, Nanjing Drum Tower Hospital, Nanjing 211800, China

## Abstract

**Aim:**

Since the high cost of reference trastuzumab limits its clinical application, this study aimed to compare the effectiveness and safety of the Zercepac and reference product trastuzumab in neoadjuvant therapy for HER2-positive breast cancer.

**Methods:**

This study retrospectively collected clinical data of patients with early-stageHER2-positive breast cancer, who received trastuzumab, pertuzumab, docetaxel, and platinum as neoadjuvant therapy from November 2020 to July 2021. Patients were divided into the Zercepac and reference trastuzumab groups. Reduction in tumor size, clinical response based on RECIST1.1 criteria, pathological complete response (pCR), and adverse events (AEs) were evaluated. Multivariate logistic regression analyses were used to adjust confounders.

**Results:**

A total of 105 patients were included in the study, among them, 65 were in the Zercepac group and 40 were in the reference trastuzumab group. The percentage of tumor shrinkage from baseline was comparable between the Zercepac and reference trastuzumab group (47.6 ± 18.6% vs. 43.0 ± 19.9%, *p* = 0.235). Clinical partial response rate was similar between the two groups (81.5% vs. 70.0%, *p* = 0.172). There were 28 cases of pCR (70.0%) in the reference trastuzumab group and 46 cases of pCR (70.8%) in the Zercepac group (*p* = 0.933). The choice of Zercepac or reference trastuzumab was not significantly associated with pCR (OR = 0.96, 95%CI: 0.41-2.28, *p* = 0.933). Adverse events (AEs) were observed in all patients, and the incidence of ≥3 grade AEs was comparable between the two groups (81.5% vs. 70.0%, *p* = 0.172).

**Conclusion:**

Zercepac has similar effectiveness and safety profile compared with reference trastuzumab in neoadjuvant therapy, which provides treatment options for patients with HER2-positive breast cancer.

## 1. Introduction

Breast cancer is the most frequently diagnosed cancer and an important cause of premature mortality among women worldwide, comprising up to 25% of all women cancers [1]. Although the incidence of breast cancer is lower in Asia (one out of 35 women, compared to one out of 8 women in the United states), it is predicted to increase in the near future [2, 3]. With breast cancer mortality rate increasing in the world during the past 25 years, prognosis of these patients significantly depends on the availability of treatment [4]. A large number of clinical studies have shown that neoadjuvant therapy significantly improves the chance of achieving high complete pathological response (pCR) rate and disease-free survival (DFS) rate, as well as overall survival (OS), of early breast cancer patients [5–7].

With the introduction of personalized treatment, the molecular biomarkers became important predictors and prognostic indicators of the therapy response in breast cancer patients [8, 9]. Trastuzumab is a human monoclonal antibody targeting HER2, that induces antibody-dependentcell-mediated cytotoxicity and inhibits its signal transduction [10]. Previous studies have proved that neoadjuvant therapy using reference trastuzumab has a significant effect on the prognosis of early-stage [11, 12] and metastatic HER2-positive breast cancer [13–15]. However, it also showed a certain cardiotoxicity in previous trials; thus, the safety and tolerability of Herceptin needs further attention [16, 17].

In many countries, the cost of trastuzumab therapy is not covered by insurance companies. High cost limits its availability for a large group of patients, who may in turn benefit from the usage of HER2 targeting therapy [18]. Zercepac is the first Chinese monoclonal antibody highly similar to reference trastuzumab, with a good application potential in reducing tumor cell proliferation and survival [19, 20]. Recently, Zercepac demonstrated efficacy equivalent to reference trastuzumab for HER2-positive recurrent or metastatic breast cancer in a phase III multicenter clinical trial [21]. However, more studies are needed to evaluate its potential efficacy in neoadjuvant therapy as well as its safety profile. More importantly, the two specification dosage forms are more suitable for Chinese patients, which can reduce the waste of residual fluid. At the same time, Zercepac does not contain preservatives, making it safe to use immediately after dispensing.

This study retrospectively collected the data of patients with HER2-positive breast cancer and evaluated the effectiveness and safety of Zercepac and reference trastuzumab in neoadjuvant therapy. This study will provide evidence for the selection of treatment and intervention strategies in breast cancer research and clinical practice.

## 2. Methods

### 2.1. Study Design and Patients

This retrospective cohort study included HER2-positive breast cancer patients who were admitted to the Breast Center of Gulou Hospital, School of Medicine, Nanjing University from November 2020 to July 2021 and received neoadjuvant therapy with trastuzumab (Zercepac or reference trastuzumab). The patients included in the study were all women under 75 years of age, with invasive breast cancer confirmed by histopathology in stages IIA, IIB, or IIIA (TNM staging according to the breast cancer AJCC guidelines [7th edition]). The immunohistology test results of HER2 expression for all patients were classified into HER-2+++, HER-2++ with ISH-positive, and HER-2+. The excluded patients were patients with obvious liver and kidney dysfunction, patients with severe cardiovascular system diseases, and patients with unstable vital signs or cachexia.

This study was approved by the Ethics Committee of Gulou Hospital, School of Medicine, Nanjing University (2021-435-02). The informed consent was waived by the ethic committee due to the retrospective nature of the study.

### 2.2. Neoadjuvant Therapy

All patients underwent standard 6 cycles of neoadjuvant chemotherapy, and the chemotherapy regimen was TCbHP regimen, which consisted of trastuzumab, pertuzumab, docetaxel, and platinum. Patients were divided into the Zercepac and reference trastuzumab groups, depending on the usage of trastuzumab: Zercepac (Shanghai Fuhong Henlius Biopharmaceutical Co Ltd) or reference trastuzumab (Roche Pharma (Schweiz) Ltd).

### 2.3. Data Collection

Clinical data such as name, age, gender, body mass index (BMI), menstrual status, molecular and histologic classification, clinical stage, tumor size, location, and metastatic lymph nodes were collected. The images of magnetic resonance imaging (MRI) scanning before and after neoadjuvant therapy were extracted. Laboratory tests included routine blood test and liver function which were obtained from the medical records as well.

### 2.4. Outcomes

Effectiveness outcomes included the shrinkage of tumor from baseline, clinical response evaluated by MRI scanning according RECIST1.1 criteria, and pathological response (pCR) rate. The Miller–Payne system used in the study has 5 grades: grade 5 is a pCR in breast; grades 1-4 are partial pathological responses according to tumor reduction ratio; from G4 to G1, the degree of tumor reduction gradually decreases [22]. Adverse events were evaluated according to the Common Terminology Criteria for Adverse Events (CTCAE 5.0).

### 2.5. Statistical Analysis

SPSS 26.0 software (IBM, Armonk, NY, USA) was used for data analysis. Continuous variables were presented as mean ± standard deviation (SD), and categorical variables were presented as frequency (percentage). For comparison between two groups, independent Student's *t*-test and *χ*2 test were used. The cutoff of continuous variables associated with pCR was determined by receiver operating characteristic (ROC) curve, optimal Youden Index, and area under the curve (AUC). Univariate and multivariate analyses were performed using the logistic regression model to evaluate prognostic factors. *p* < 0.05 indicated a statistical significance.

## 3. Results

### 3.1. Baseline Characteristics

A total of 105 patients with HER2-positive breast cancer received neoadjuvant therapy and were included in the study. Among these patients, 65 patients were in the Zercepac group and 40 patients were in the reference trastuzumab group. The mean age was 48.5 ± 9.3 and 49.2 ± 8.9 years in the Zercepac and reference trastuzumab groups, respectively. The detailed baseline characteristics are shown in Table 1.

### 3.2. Effectiveness

**Figure 1:**
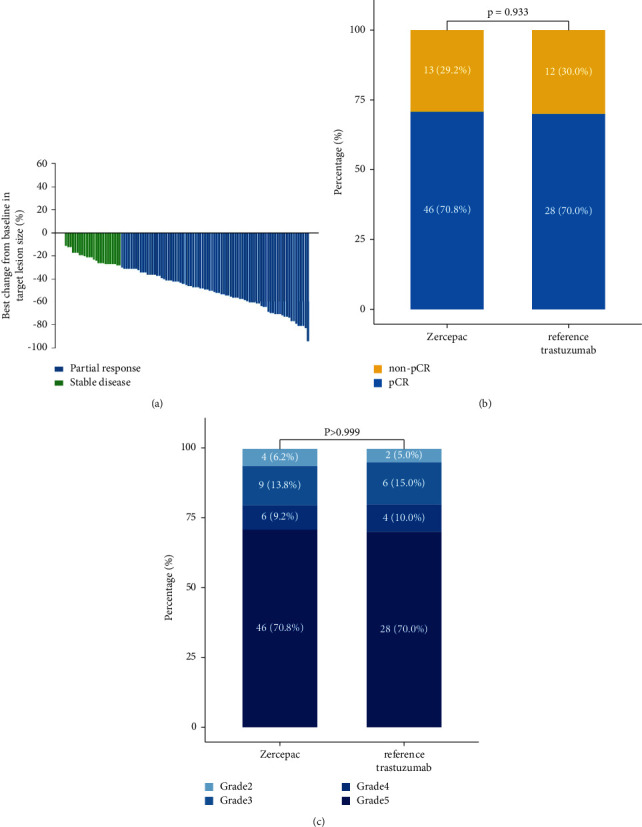
Treatment response.

**Figure 2:**
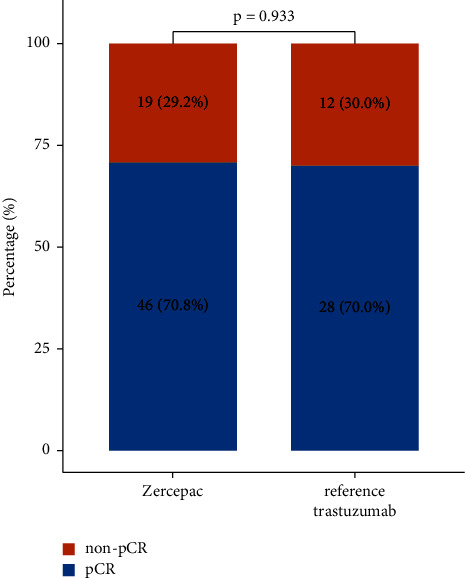
Comparison of complete pathological remission (pCR) rate in patients who received reference trastuzumab and Zercepac.

**Figure 3:**
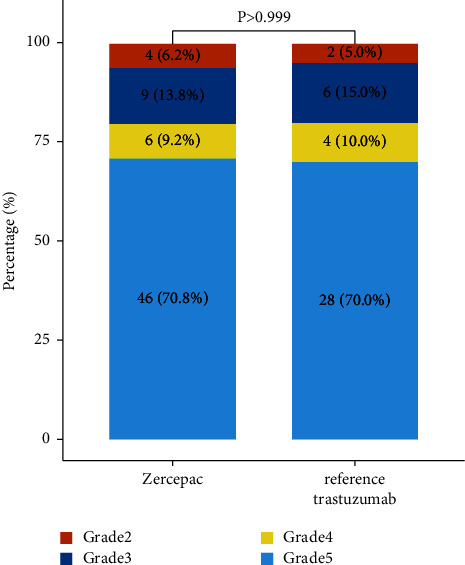
Detailed pathological response to treatment in patients who received reference trastuzumab and Zercepac.

Treatment response of individual patients is shown in Figure 1. The percentage of shrinkage of tumor from baseline was comparable between the Zercepac and reference trastuzumab groups (47.6 ± 18.6% vs. 43.0 ± 19.9%, *p* = 0.235, Table 2). Clinical partial response rate was similar between the two groups (81.5% vs. 70.0%, *p* = 0.172). Regarding pathological response to neoadjuvant therapy, there were 28 cases of pCR (70.0%) and 12 cases of non-pCR (30.0%) in the reference trastuzumab group, while in the 46 cases achieved pCR in the Zercepac group (70.8%) with no significant difference (*p* = 0.933, Figure 2). Among the non-pCR patients, in the Zercepac group Grade 2 partial response was reported in 6.2%, Grade 3 was reported in 13.8%, and Grade 4 was reported in 9.2% of patients. In reference trastuzumab group, Grade 2 partial response was reported in 5.0%, Grade 3 in 15.0%, and Grade 4 in 10.0% of patients (Figure 3). There was no statistically significant difference between the two groups (all *p* > 0.05).

### 3.3. Predictors of Response

For maximum tumor diameter, neutrophil-to-lymphocyte ratio (NLR), platelet-lymphocyte ratio (PLR), and Ki67 expression, ROC curves and optimal Youden Index were used to discriminate the cutoff value (Supplemental Figure 1).

Multivariate analyses entering maximum tumor diameter, NLR, PLR, and Ki67 expression as continuous or categorical variables were carried out. The choice of Zercepac or reference trastuzumab was not significantly associated with pCR (OR = 0.96, 95%CI: 0.41-2.28, *p* = 0.933). In the meantime, progesterone receptors status (OR = 5.21, 95%CI: 1.64-16.55, *p* = 0.005), maximum tumor diameter (OR = 0.13, 95%CI: 0.05-0.38, *p* < 0.001), and NRL (OR = 0.22, 95%CI: 0.12-0.42, *p* < 0.001) were all independent predictors of response (Table 3).

### 3.4. Safety

The incidence rate of AEs was both 100.0% in the Zercepac and reference trastuzumab groups (Table 4). The most common AEs in the Zercepac group were hair loss (89.2%), elevated AST (aspartate aminotransferase, 69.2%), and fatigue (67.7%), while the most common in the reference trastuzumab group were hair loss (87.5%), decreased appetite (70.0%), and elevated ALT (alanine aminotransferase, 70.0%).

Grades ≥3 AEs were reported in 53 (81.5%) and 28 (70.0%) patients in the Zercepac and reference trastuzumab groups (*p* = 0.172), respectively. The most common grades ≥3 AEs in the Zercepac group were elevated AST (29.2%), anemia (24.6%), and elevated ALT (23.1%), while the most common grades ≥3 AEs in the reference trastuzumab group were insomnia (22.5%), fatigue (15.0%), and evaluated AST (15.0%). The incidence of grade ≥3 insomnia and fatigue was significantly higher in the reference trastuzumab group than those in the Zercepac group (both *p* < 0.05).

## 4. Discussion

This retrospective cohort study compared the effectiveness and safety of Zercepac and reference trastuzumab as neoadjuvant therapy in patients with early-stageHER2-positive breast cancer. The findings of this study demonstrated that Zercepac and reference trastuzumab resulted in a comparable pCR rate, which was further confirmed by multivariate analysis. Regarding adverse events, both regimens showed similar safety profile.

In recent years, with the continuous innovation of medical technology, many breast cancer trials are focused on the tumor-promoting gene HER2, which has an impact on cell reproduction, differentiation, and survival [23]. Trastuzumab is a humanized monoclonal antibody derived from recombinant DNA that can selectively suppress the expression of HER2 protein in HER2-positive cells [24, 25]. Previous studies have confirmed the efficacy of reference trastuzumab in patients with HER2-positive metastatic breast cancer combined with chemotherapy [15, 26] and neoadjuvant therapy [11, 27]. Patent expirations for trastuzumab in the European Union (2014) and USA (2019) have led the development of several bioequivalent drugs with low development costs [28]. In the neoadjuvant setting, both biosimilar SB3 and CT-P6 were equivalent to reference trastuzumab with respect to pCR (SB3: 51.7% and 42.0%; CT-P6: 46·8% and 52.4%) in phase 3, randomized trials [29, 30]. In real-world studies, biosimilar CT-P6 demonstrated pCR comparable to reference trastuzumab (CT-P6: 74.4% vs. 69.8%, *p* = 0.411) in HER2-positiveearly-stage breast cancer [31], while SB3 showed a pCR rate comparable to that seen in previous clinical studies [32]. Consistently, in this retrospective study, the pCR rate was similar in the reference trastuzumab and Zercepac groups (70.8% vs. 70.0%), and further multivariate logistic regression showed the choice of Zercepac or reference trastuzumab was not significantly associated with pCR. Moreover, the percentage of tumor shrinkage and clinical response was comparable between the reference trastuzumab and Zercepac groups. Based on the results of the abovementioned studies, it is suggested that the effectiveness of Zercepac was comparable to that of reference trastuzumab in HER2-positive breast cancer in the neoadjuvant setting.

Reference trastuzumab and its biosimilars were shown to be relatively well tolerated [33]. In the phase 3 clinical trial of trastuzumab SB3, no difference in the incidence of AEs was observed between SB3 and reference trastuzumab (96.6% and 95.2%), with the most common AEs being neutropenia and alopecia [29]. In the randomized clinical trial of trastuzumab CT-P6, the incidence of AEs in patients with HER2-positive breast cancer was comparable between CT-P6 and reference trastuzumab (94% and 95%), while the most commonly reported serious AEs were febrile neutropenia and neutropenia [30]. In this study, though the AEs occurred in all patients in the Zercepac group, grade ≥3 AEs were mostly common AEs of chemotherapy, which were manageable and draw no additional safety concern. Furthermore, the incidence rates of AEs and grade ≥3 AEs were comparable between the Zercepac and reference trastuzumab group. The similar safety profile demonstrated that patients can well tolerate both reference trastuzumab and Zercepac.

This study has the following limitations. Firstly, due to the retrospective design, data loss or ambiguity might occur in the process of data collection. Secondly, this study does not involve follow-up, and the long-term survival rate and quality of life of patients are not included in the results. Whether or not the observed difference in the tumor size reduction would result in different PFS is not known and should be investigated in future studies.

## 5. Conclusion

In conclusion, the effectiveness and safety of Zercepac were comparable to that of reference trastuzumab in HER2-positive breast cancer when administered in the neoadjuvant setting with pertuzumab, docetaxel, and platinum. This study could provide evidence for the application of domestic trastuzumab Zercepac in the clinical practice, which might contribute to the possibility of anti-HER2 therapy available to a wider range of patients.

## Figures and Tables

**Table 1:** Baseline characteristics.

Characteristic	Total (*N* = 105)	Zercepac (*n* = 65)	Reference trastuzumab (*n* = 40)	*P*
Age, years, mean ± SD	48.73 ± 9.11	48.48 ± 9.28	49.15 ± 8.93	0.715

Sex, *n* (%)				
Female	105 (100.0)	65 (100.0)	40 (100.0)	
Male	0	0	0	

BMI, kg/m^2^, median (range)	23.20 (19.40, 26.50)	22.40 (19.40, 26.50)	23.40 (19.62, 26.47)	0.877

Menstruation situation, *n* (%)				0.658
Premenopausal	47 (44.8)	28 (43.1)	19 (47.5)	
Postmenopausal	58 (55.2)	37 (56.9)	21 (52.5)	

Histological typing, *n* (%)				
Invasive carcinoma	105 (100.0)	65 (100.0)	40 (100.0)	
Other	0	0	0	

Histological grading, *n* (%)				0.060^*∗*^
Grade 1	5 (4.8)	3 (4.6)	2 (5.0)	
Grade 2	35 (33.3)	27 (41.5)	8 (20.0)	
Grade 3	65 (61.9)	35 (53.8)	30 (75.0)	

Time from diagnosis to trastuzumab treatment, days, media (range)	6 (4, 9)	6 (4, 12)	6 (4, 8)	0.691

HER2 status, *n* (%)				
-	0	0	0	
+	105 (100.0)	65 (100.0)	40 (100.0)	

ER status, *n* (%)				0.728
-	45 (42.9)	27 (41.5)	18 (45.0)	
+	60 (57.1)	38 (58.5)	22 (55.0)	

PR status, *n* (%)				0.059
-	67 (63.8)	46 (70.8)	21 (52.5)	
+	38 (36.2)	19 (29.2)	19 (47.5)	

Molecular typing, *n* (%)				0.799
HER2+HR-	41 (39.0)	26 (40.0)	15 (37.5)	
HER2+HR+	64 (61.0)	39 (60.0)	25 (62.5)	

Ki67, %, median (range)	40.00 (40.00, 60.00)	50.00 (40.00, 60.00)	40.00 (30.00, 50.00)	0.026
Maximum tumor diameter, mm, median (range)	24.00 (18.00, 33.00)	24.00 (18.00, 34.00)	22.00 (18.75, 32.00)	0.815
NLR, median (range)	2.67 (1.78, 3.56)	2.57 (1.88, 3.56)	2.72 (1.64, 3.48)	0.779
PLR, median (range)	179.41 (137.73, 219.43)	174.61 (137.28, 218.39)	191.96 (139.06, 228.33)	0.409

BMI—Body mass index; HER2—human epidermal growth factor receptor 2; ER—estrogen receptors; PR—progesterone receptors; NLR—neutrophil-to-lymphocyte ratio; PLR—platelet-lymphocyte ratio; SD—standard deviation.

**Table 2:** Effectiveness.

	Total (*N* = 105)	Zercepac (*n* = 65)	Reference trastuzumab (*n* = 40)	*p*
Percentage of tumor shrinkage from baseline, %	45.84 ± 19.15	47.58 ± 18.60	43.00 ± 19.92	0.235

Clinical response, *n* (%)				0.172
PR	81 (77.1)	53 (81.5)	28 (70.0)	
SD	24 (22.9)	12 (18.5)	12 (30.0)	

Pathological response, *n* (%)				0.933
Non-pCR	31 (29.5)	19 (29.2)	12 (30.0)	
pCR	74 (70.5)	46 (70.8)	28 (70.0)	

MP grade, *n* (%)				>0.999^*∗*^
Grade 2	6 (5.7)	4 (6.2)	2 (5.0)	
Grade 3	15 (14.3)	9 (13.8)	6 (15.0)	
Grade 4	10 (9.5)	6 (9.2)	4 (10.0)	
Grade 5	74 (70.5)	46 (70.8)	28 (70.0)	

PR—partial response; SD—stable disease; pCR—complete pathological response.

**Table 3:** Univariate and multivariate analyses of factors associated with complete pathological response.

Characteristics	Univariate analysis		Multivariate analysis 1^*∗*^		Multivariate analysis 2^*∗*^^*∗*^	
	OR (95%CI)	*p*	OR (95%CI)	*p*	OR (95%CI)	*p*
Age	1.02 (0.98-1.07)	0.327				

BMI	0.98 (0.88-1.08)	0.646				

Menstruation situation						
Premenopausal	1					
Postmenopausal	0.70 (0.40-1.65)	0.420				

Histological grading						
Grade 1	1					
Grade 2	6.00 (0.84-43.09)	0.075				
Grade 3	3.14 (0.49-20.25)	0.228				

Time from diagnosis to trastuzumab treatment	1.11 (1.00-1.23)	0.053				

ER status						
-	1					
+	2.00 (0.85-4.66)	0.111				

PR status						
-	1		1		1	
+	3.18 (1.17-8.65)	0.024	4.65 (0.86-25.27)	0.075	5.21 (1.64-16.55)	0.005

Molecular typing						
HER2+HR-	1					
HER2+HR-	2.09 (0.89-4.91)	0.090				

Ki67	1.02 (0.99-1.05)	0.171				

Ki67 (ROC cutoff)						
≤40%	1					
>40%	2.26 (0.95-5.38)	0.065				

Maximum tumor diameter	0.93 (0.89-0.97)	<0.001	0.909 (0.85-0.97)	0.005		

Maximum tumor diameter (categorical)						
≤30 mm	1				1	
>30 mm	0.20 (0.08-0.49)	<0.001			0.14 (0.05-0.38)	<0.001

NLR	0.23 (0.13-0.41)	<0.001	0.22 (0.12-0.42)	<0.001		

NLR (categorical)						
≤2.68	1					
>2.68	0.00 (0.00-Inf)	0.989				

PLR	1.00 (0.99-1.01)	0.656				

PLR (categorical)						
≤189.33	1					
>189.33	1.82 (0.77-4.32)	0.176				

Trastuzumab						
Zercepac	1					
Reference trastuzumab	0.96 (0.41-2.28)	0.933				

^*∗*^Continuous variable was entered as continuous variable. ^*∗∗*^Continuous variable was entered as categorical variables using optimal Youden Index-based cutoff point. OR—odds ratio; BMI—body mass index; HER2—human epidermal growth factor receptor 2; ER—estrogen receptors; PR—progesterone receptors; NLR—neutrophil-to-lymphocyte ratio; PLR—platelet-lymphocyte ratio.

**Table 4:** Safety.

	All grades			Grades ≥3		
	Zercepac (*n* = 65)	Reference trastuzumab (*n* = 40)	*p*	Zercepac (*n* = 65)	Reference trastuzumab (*n* = 40)	*p*
Any AE	65 (100.0)	40 (100.0)	—	53 (81.5)	28 (70.0)	0.172
Nausea	28 (43.1)	16 (40.0)	0.756	6 (9.2)	3 (7.5)	0.758
Vomit	33 (50.8)	17 (42.5)	0.410	9 (13.8)	4 (10.0)	0.561
Fatigue	44 (67.7)	23 (57.5)	0.291	2 (3.1)	6 (15.0)	0.025
Infusion-related reaction	2 (3.1)	15 (37.5)	<0.001	0	1 (2.5)	0.200
Diarrhea	36 (55.4)	17 (42.5)	0.200	5 (7.7)	5 (12.5)	0.415
Decreased appetite	31 (47.7)	28 (70.0)	0.025	3 (4.6)	4 (10.0)	0.283
Elevated AST	45 (69.2)	26 (65.0)	0.653	19 (29.2)	6 (15.0)	0.096
Elevated ALT	36 (55.4)	28 (70.0)	0.136	15 (23.1)	5 (12.5)	0.180
Elevated GGT	12 (18.5)	11 (27.5)	0.277	1 (1.5)	0 (0.0)	0.431
Anemia	33 (50.8)	22 (55.0)	0.673	16 (24.6)	5 (12.5)	0.132
Thrombocytopenia	32 (49.2)	13 (32.5)	0.093	5 (7.7)	2 (5.0)	0.591
Hypertriglyceridemia	14 (21.5)	17 (42.5)	0.022	3 (4.6)	5 (12.5)	0.139
Hypokalemia	3 (4.6)	0	0.168	1 (1.5)	0 (0.0)	0.431
Neutropenia	20 (30.8)	3 (7.5)	0.005	1 (1.5)	0 (0.0)	0.431
Leukopenia	30 (46.2)	11 (27.5)	0.057	2 (3.1)	3 (7.5)	0.301
Infection	7 (10.8)	2 (5.0)	0.305	0	0	—
Dizziness	26 (40.0)	15 (37.5)	0.799	7 (10.8)	1 (2.5)	0.121
Hectic fever	22 (33.8)	15 (37.5)	0.703	10 (15.4)	4 (10.0)	0.431
Difficulty breathing	5 (7.7)	2 (5.0)	0.591	0	0	—
Rash	13 (20.0)	17 (42.5)	0.013	1 (1.5)	1 (2.5)	0.726
Myalgia	23 (35.4)	25 (62.5)	0.007	2 (3.1)	0 (0.0)	0.263
Cough	7 (10.8)	9 (22.5)	0.104	0	2 (5.0)	0.069
Hair loss	58 (89.2)	35 (87.5)	0.787	0	0	—
Insomnia	17 (26.2)	23 (57.5)	0.001	0	9 (22.5)	<0.001

AST—aspartate aminotransferase; ALT—alanine aminotransferase; GGT—gamma-glutamyl transferase.

## Data Availability

The datasets supporting the results of this article are included within the article and supplementary materials. Other datasets used and/or analyzed during the current study are available from the corresponding author on reasonable request.

## Supplementary Materials

Supplementary Materials Supplementary Figure 1. Receiver operating characteristic (ROC) curves used to discriminate the cutoff of Ki67, maximum tumor diameter, neutrophil-to-lymphocyte ratio (NLR), and platelet-lymphocyte ratio (PLR) when predicting complete pathological remission. Cutoff value was determined using Youden Index.
