# Supplementary material for: Effectiveness and Safety of Zercepac and Reference Trastuzumab in the Neoadjuvant Setting for Early-Stage Breast Cancer: A Retrospective Cohort Study
Source: J Oncol. 2022 Nov 3;2022:9998114. doi: 10.1155/2022/9998114 (PMC9649327; doi:10.1155/2022/9998114)
Supplement: Supplementary Materials — Supplementary Figure 1. Receiver operating characteristic (ROC) curves used to discriminate the cutoff of Ki67, maximum tumor diameter, neutrophil-to-lymphocyte ratio (NLR), and platelet-lymphocyte ratio (PLR) when predicting complete pathological remission. Cutoff value was determined using Youden Index. [file 9998114.f1.docx]

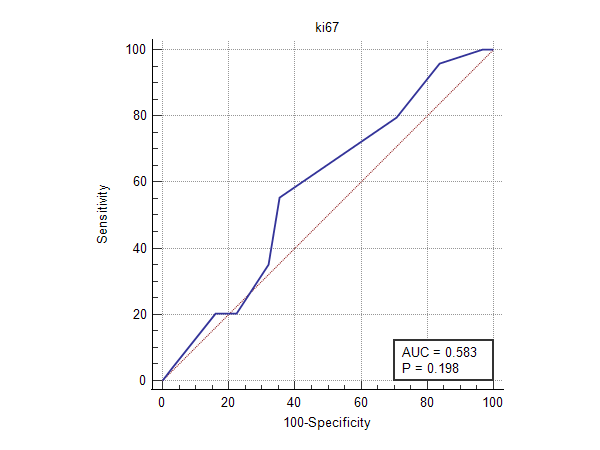

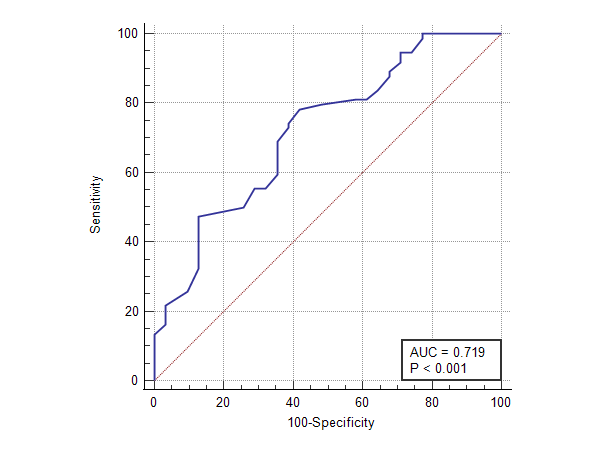

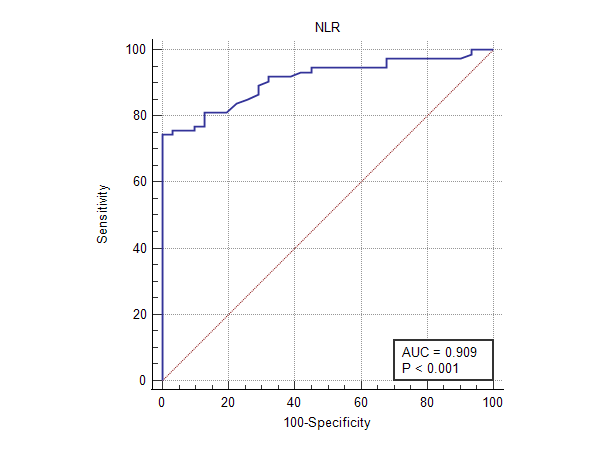

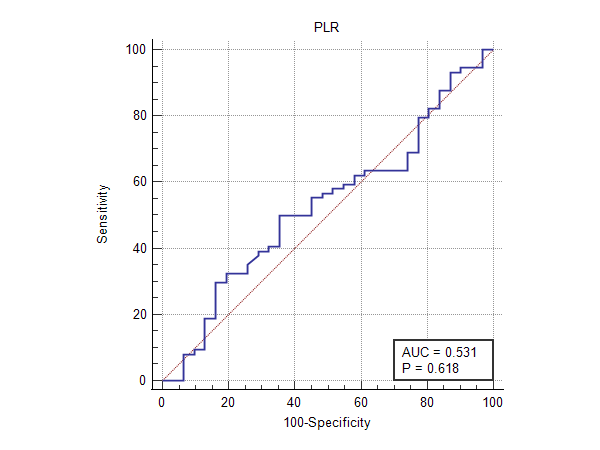


|  | Cut-off | AUC (95% CI) | Sensitivity (95% CI) | Specificity (95% CI) |
| --- | --- | --- | --- | --- |
| ki67 | >40 | 0.583 (0.482-0.678) | 55.4 (43.4-67.0) | 64.5 (45.4-80.8) |
| Maximum tumor diameter | >30 | 0.719 (0.623-0.802) | 78.4 (67.3-87.1) | 58.1 (39.1-75.5) |
| NLR | >2.68 | 0.909 (0.837-0.956) | 74.3 (62.8-83.8) | 100 (88.0-100.0) |
| PLR | >189.33 | 0.531 (0.431-0.629) | 50.0 (38.1~61.9) | 64.5 (45.4-80.8) |

**Supplementary Figure 1.** Receiver operating characteristic (ROC) curves used to discriminate the cut-off of Ki67, maximum tumor diameter, neutrophil to lymphocyte ratio (NLR) and platelet-lymphocyte ratio (PLR) when predicting complete pathological remission. Cut-off value was determined with Youden index.
